# Feasibility and potential effects of a combined money advice and psychological therapy intervention within National Health Service Talking Therapies services

**DOI:** 10.1192/bjo.2025.37

**Published:** 2025-06-11

**Authors:** Hannah Louise Belcher, Lois Parri, Imogen Kilcoyne, Joanne Evans, Caroline Da Cunha Lewin, Robin Lau, Nicola Bond, Conor D’Arcy, Melissa Hatch, Til Wykes

**Affiliations:** Institute of Psychiatry, Psychology & Neuroscience, King’s College London, London, UK; The Money and Mental Health Policy Institute, The Policy Institute at King’s, London, UK; Department of Health Service & Population Research, Citizens Advice, London, UK

**Keywords:** Anxiety, cognitive–behavioural therapy, debt, money advice, talking therapies

## Abstract

**Background:**

It is now accepted that social factors affect not only onset but also mental health treatment outcomes. One such factor is financial difficulty. Within National Health Service (NHS) Talking Therapies, problem debt has been shown to interfere significantly with recovery from mental health problems, estimated as 22% versus 50% recovered with no problem debt. One solution is a combined money advice and psychological therapy intervention to improve treatment outcomes.

**Aims:**

The aim of the current study was to trial a combined money advice and psychological therapy service within NHS Talking Therapies, to ascertain its feasibility and acceptability.

**Methods:**

This study employed a mixed methods case series of individuals attending high-intensity cognitive–behavioural treatment who were provided with a combined intervention (money advice service plus NHS Talking Therapies). Acceptability and feasibility were evaluated through interviews, and benefit was assessed from comparisons of routinely collected symptom measures and compared to historical recovery estimates.

**Results:**

Some 32 participants, with similar gender distribution but more representation from ethnic minorities, were recruited from NHS Talking Therapies. One-third demonstrated complete recovery on both depression and anxiety, while half showed symptom improvement and modest improvements on the financial outcomes measure. Our interviews with patients, therapists and money advisors suggested the combined intervention was acceptable and beneficial, but that money worries should be identified earlier.

**Conclusions:**

The combined service is acceptable, accessible and could deliver benefit, even in the short term, to those with mental health and debt problems.

We know that social factors can predict or prevent the onset of a disorder but rarely are they considered in understanding treatment outcomes.^1^ However, if these factors are important to the onset and potentially the maintenance of symptoms, they should be an important consideration in developing the treatment protocol. One of these factors is financial distress. We know that there is a bi-directional link between money worries and mental health difficulties. Money worries exacerbate mental health problems, and mental health problems lead to more financial problems.^2–5^ Evidence is also emerging of a link between financial difficulties and suicidality^6^ and self-harm.^7^ With the cost-of-living crisis pushing many UK households into debt,^8^ there is the potential for increasing mental health problems. Some individuals are also more vulnerable to financial difficulties than others, for example, those who are single, female and from minoritised households.^9,10^ It has been suggested that money advice should be provided alongside psychological therapy to aid recovery.^11^

Evidence to support the potential for money advice to improve recovery comes from a variety of sources. Citizens Advice, the leading UK charity offering financial and legal advice, reported fewer symptoms of anxiety and depression after receiving money advice,^12^ and the McPin Foundation, a UK mental health research charity, found that website information plus a telephone advice line improved well-being.^13^ However, there are potential difficulties in accessing such services. For instance, individuals may feel too overwhelmed by their difficulties, and struggle to remember and initiate the advice given.^14^ Therefore, a collaborative approach, where psychological therapists work alongside debt advisers to address patients’ difficulties, may be more beneficial^11^ and a small pilot in emergency departments in those presenting with suicidal thoughts or self-harm found this sort of combined service feasible and acceptable.^15^ However, it is not yet clear whether a combined money advice and psychological therapy intervention is feasible or what the potential benefits would be.

The UK mental health service treating people with common mental health problems is the National Health Service (NHS) Talking Therapies service (formally known as Improving Access to Psychological Therapies, IAPT), which has over one million clients each year with mild to moderate anxiety and depression.^16^ Their stepped care model offers low-intensity (guided self-help) and high-intensity interventions (one-to-one cognitive–behavioural therapy). There is also evidence that recovery rates are affected by deprivation,^1^ and Acton^14^ predicted that recovery rates could be as low as 22% for those with depression and financial difficulties, versus 55% for those without financial difficulties, with similar figures for anxiety (38% and 52%, respectively). When asked about a combined intervention based in NHS Talking Therapies, patients, NHS therapists and Citizens Advice money advisors thought this would be helpful as more holistic care provided an easier referral pathway, making the money advice more accessible and mutual agency support beneficial.^17^ Therapists also suggested that it would protect therapeutic time, as often a patient’s money worries became the session focus.

To assess the feasibility and potential benefit of a combined intervention, we assessed the combined intervention in an area of high deprivation supported by three NHS Talking Therapies services. We based our questions on Orsmand and Cohn’s^18^ recommendations for pilot studies: to assess participant responses to the intervention, and its potential effectiveness as well as recruitment capability, data collection and outcomes. The most important issue for any rollout of a combined service is the eventual take-up, as that affects resourcing and the overall costs.

## Method

### Study design

A mixed methods case series of individuals referred for high-intensity intervention in the Talking Therapies services in three London boroughs who reported financial difficulties, particularly problem debt, was used. A direct pathway was set up with Citizens Advice, so that participants could be directly referred to a dedicated money advisor. These money advice sessions followed Citizens Advice’s standard service, where an advisor meets the client to discuss what they would like help with, and then makes a plan with the client on how they can best be supported. This may involve helping them with benefit applications, managing debts and/or budgeting finances. The decisions on which services and issues are the most important are therefore made on an individual collaborative basis but did not include legal representation. Demographic characteristics were collected at baseline, and Talking Therapies routinely collected outcomes on depression, anxiety and functioning (Patient Health Questionnaire – 9 (PHQ-9), Generalised Anxiety Disorder – 7 (GAD-7) questionnaire and Work and Social Adjustment Scale – 5 (WSAS-5)), assessed at baseline and discharge. Recovery was assessed using Talking Therapies metrics^19^ and compared with national data on estimated recovery effects for those with mental health and financial difficulties. In addition, we measured general improvement on the routinely collected measures. A financial outcome was collected only at the end of the psychological intervention. Qualitative data on the process and experience of therapy and money advice were also collected at the end of the psychological therapy through exit interviews. Therapists and money advisors were interviewed to gain their perspectives on the challenges and benefits of the combined treatment.

Ethical approval: The authors assert that all procedures contributing to this work comply with the ethical standards of the relevant national and institutional committees on human experimentation and with the Helsinki Declaration of 1975, as revised in 2013. All procedures involving patients were approved by Cambridge East Research Ethics Committee NHS (21/EE/0159). All participants were provided with an information sheet, including signposting to organisations that provide support for mental health and financial difficulties, and written consent was given before the study. Trial registration: NCT04926675.

### Recruitment and eligibility criteria

Participants were eligible to take part in the study and intervention if they had begun, or were about to begin, high-intensity one-to-one psychological therapy in one of three boroughs within the South London and Maudsley NHS Foundation Trust (Lambeth, Lewisham and Southwark) and were identified as having problem debt or money difficulties that was affecting their mental health. No exclusion criteria were included, and translators were provided for those who were unable to communicate in English. Suitable referrals were given study information, the study team ascertained their consent and the therapist then referred them to the money advice service within Citizens Advice.

### Developing the combined money advice and psychological therapy intervention

The combined intervention procedure was developed from interviews with Talking Therapies clients, therapists and money advisors.^17^ The money advice sessions were designed for patients’ needs, with no limit on number of sessions. There was a preference for money advice and psychological treatment to be provided in parallel to aid mutual agency support; however, if necessary, money advice could continue after therapy ended. Collaboration between money advisors and therapists was acceptable to participants, with information being shared across both services to ensure participants received the appropriate care and support.

### Outcome measures

#### Routine outcome measures from NHS Talking Therapies

PHQ-9: Measuring depression using DSM-IV criteria^20^ with items rated on a 4-point scale from ‘not at all’ to ‘every day’. Scores range from 0 to 27, with clinical cases scoring 10 or above.

GAD-7: Measuring anxiety using DSM-IV criteria^21^ with items rated on a 4-point scale from ‘not at all’ to ‘every day’. Scores range from 0 to 21, with clinical cases scoring 8 or above.

WSAS-5:^22^ Measuring impaired functioning associated with mental disorders^22^ using five items rated on an 8-point scale from ‘not at all’ to ‘very severely’. Scores between 10 and 19 indicate moderate impairment, and 20–40 indicate severe impairment.

Financial confidence assessed at the exit interview: a patient advisory group (the National Institute for Health and Care Research (NIHR) Maudsley Biomedical Research Centre’s (BRC’s) Feasibility and Acceptability Support Team for Researchers (FAST-R)) chose the most important questions from the client audit survey used by Citizens Advice. These questions related to the following:

- improvements in control over finances;
- a more secure housing situation;
- feeling less stressed, depressed or anxious;
- found it easier to do or find a job;
- had better relationships and friendships;
- found it easier to get on with their day-to-day life.

Questions were rated on a 5-point scale from agree ‘not at all’ to ‘a great deal’. Not all items were relevant to every participant, so average scores were collated for each item.

#### Descriptive and context measures

Demographics including ethnicity, age, gender, disability and employment status.

Recruitment and adherence rates were measured as the number of patient participants who agreed to and fulfilled a money advice referral. Our key measure was adherence, defined as attending at least one money advice session.

#### Qualitative assessment

Semi-structured interviews with patients, therapists and money advisors were carried out at the end of the participant’s Talking Therapies treatment. Interviews were based around a topic guide (see Supplement 1). Summaries were sent to participants for checking after the interviews had been conducted.

### Data analysis

#### Quantitative analysis

Feasibility and acceptability of the service were ascertained by the number of appropriate referrals and the number of sessions of psychological treatment attended and, because the money advice service is often carried out over a lengthy period, whether the client attended at least one session.

Potential benefits of the combined intervention were initially assessed using^19^ guidelines for assessing recovery. Recovery is demonstrated if scores on the PHQ-9 and/or the GAD-7 questionnaire are above the clinical cut-off at the start of treatment (≥10 and/or ≥8, respectively) and below these clinical cut-offs at the end of treatment. We also investigated recovery on the WSAS-5 by taking a similar approach, with recovery demonstrated if their score was above the impairment threshold at the start of treatment (≥10) and below it at the end. As our sample is receiving a high level of service, we adopted the 22% threshold for recovery estimated by Acton^14^ for those with problem debt and depression or anxiety. We considered this the baseline for the combined treatment to provide some benefit. We also set symptom improvement levels as participants reducing their mental health difficulties by at least 4 points on the GAD-7 questionnaire or 5 points on the PHQ-9, which was considered to be improved.^23^

Exploratory analyses were performed to determine if any demographic group was more likely to reach reliable recovery than others, as there is evidence that some characteristics are related to higher levels of financial difficulty. These analyses employed chi-square tests with the categorical dependent variables ‘recovery’ and ‘no recovery’ and the independent variables gender (male or female), ethnicity (ethnic minority or White), disability (disabled or not) and employment (employed/other or unable to work/unemployed).

#### Qualitative analysis

Exit interviews were analysed thematically following Braun and Clarke’s^24^ method using NVivo 12 software (Lumivero, Denver, CO, USA; see https://lumivero.com/products/nvivo/). Four researchers independently analysed the data from each group of interviewees separately (therapists, advisors and patients) by first familiarising themselves with the data, then coding and generating themes. Analyses were reviewed for discrepancies by H.L.B.

#### Power of the study

This case series ascertains the potential for a new service, so to mirror the service we included individuals with different sorts of problem debt and different mental health difficulties that were serious enough to warrant high-intensity therapy. We also recruited more than the ten cases generally recommended not only to capture this diversity, as suggested by others,^25^ but also to provide an indication of potential benefits across a mixed group. We therefore extended recruitment to at least 30 cases to be able to see a signal of benefit and potential to identify if any variables were likely to affect this benefit.

#### Involvement of people with lived experience

The FAST-R service is made up of people with a variety of lived experiences. They considered the study protocol, all study information and consent, as well as the financial outcome measure. The design of the intervention was guided by interviews with those with lived experience and participants were involved in checking the analysis of the interview data. Six researchers who carried out the study, the qualitative analysis and writing the paper had lived experience and are also authors of this paper.

## Results

### Recruitment, baseline characteristics and acceptability

Seventy-three patients indicated an interest in taking part in the intervention and gave consent for us to contact them. Some 35 (48%) responded to emails and phone calls and providing consent to be referred to a money advisor. Of these, 32 (91%) attended a money advice session, with one patient in each borough not attending a money advice session (see CONSORT flow chart in the supplementary materials). There was some missing demographic information on the Talking Therapies systems; however, our sample characteristics were varied (see Table 1). From those that were recorded, 71.9% were women, 46.8% were from ethnic minority backgrounds, 53.2% were in either full-time or part-time employment, with 18.8% unemployed, 12.5% had disabilities and ages ranged from 20 to 61 (mean = 39.6). Compared to Talking Therapies’ annual statistics across all of England,^26^ the gender distribution recruited to the combined intervention was comparable (67% women); however, our sample had more individuals from minoritised communities and fewer individuals with disabilities.

**Table 1:** Participant demographics of those attending money advice and therapy

Demographics		Patients (*n* = 32)
Gender		
Female		23 (71.9%)
Male		9 (28.1%)
Ethnicity		
White British/other		13 (40.63%)
Black Caribbean		8 (25%)
Black African		4 (12.5%)
Black other		1 (3.1%)
Bangladeshi or Indian		1 (3.1%)
Latin American		1 (3.1%)
Other		2 (6.3%)
Not listed		1 (3.1%)
Disability		
Yes		4 (12.5%)
No		21 (65.6%)
Not listed		6 (18.8%)
Employment		
Employed full time		10 (31.3%)
Employed part time		7 (21.9%)
Homemaker		1 (3.1%)
Self-employed		2 (6.3%)
Student		1 (3.1%)
Unable to work		3 (9.4%)
Unemployed		6 (18.8%)
Not listed		2 (6.3%)

Most participants who took part in the combined intervention completed both baseline and end-of-treatment outcome measures taken from the Talking Therapies services (PHQ-9, GAD-7 questionnaire and WSAS-5) but three had missing data (one did not complete any measure, one had a missing baseline WSAS-5 score and one fell below the cut-off at entry on the GAD-7 questionnaire). Participants with missing data were removed from relevant parts of the quantitative analysis. Fourteen (40%) attended the exit interviews and completed the financial outcomes measure. Nearly all therapists and money advisors provided interviews for each of their clients (31 and 32, respectively).

### Potential treatment benefits

See Table 3 in the supplementary material, available at https://doi.org/10.1192/bjo.2025.37, for individual participant data.

Symptoms (*n* = 31): One-third (*n* = 10, 33.3%) recovered as measured by anxiety symptoms, two-fifths (*n* = 12, 40%) recovered as measured by depression symptoms and one-third (*n* = 10, 33.3%) demonstrated recovery on both measures (i.e. above our baseline of 22% recovery for this group). Half showed improvement in the GAD-7 questionnaire and PHQ-9.

Functioning (*n* = 31): Of patients who received money advice and completed pre- and post-WSAS-5 assessments, 20% (*n* = 4) demonstrated recovery at the end of treatment.

Secondary analyses: No analyses demonstrated that any demographic characteristic predicted recovery on either the GAD-7 questionnaire, PHQ-9 or WSAS-5 (all *p* values > .05).

**Fig. 1:**
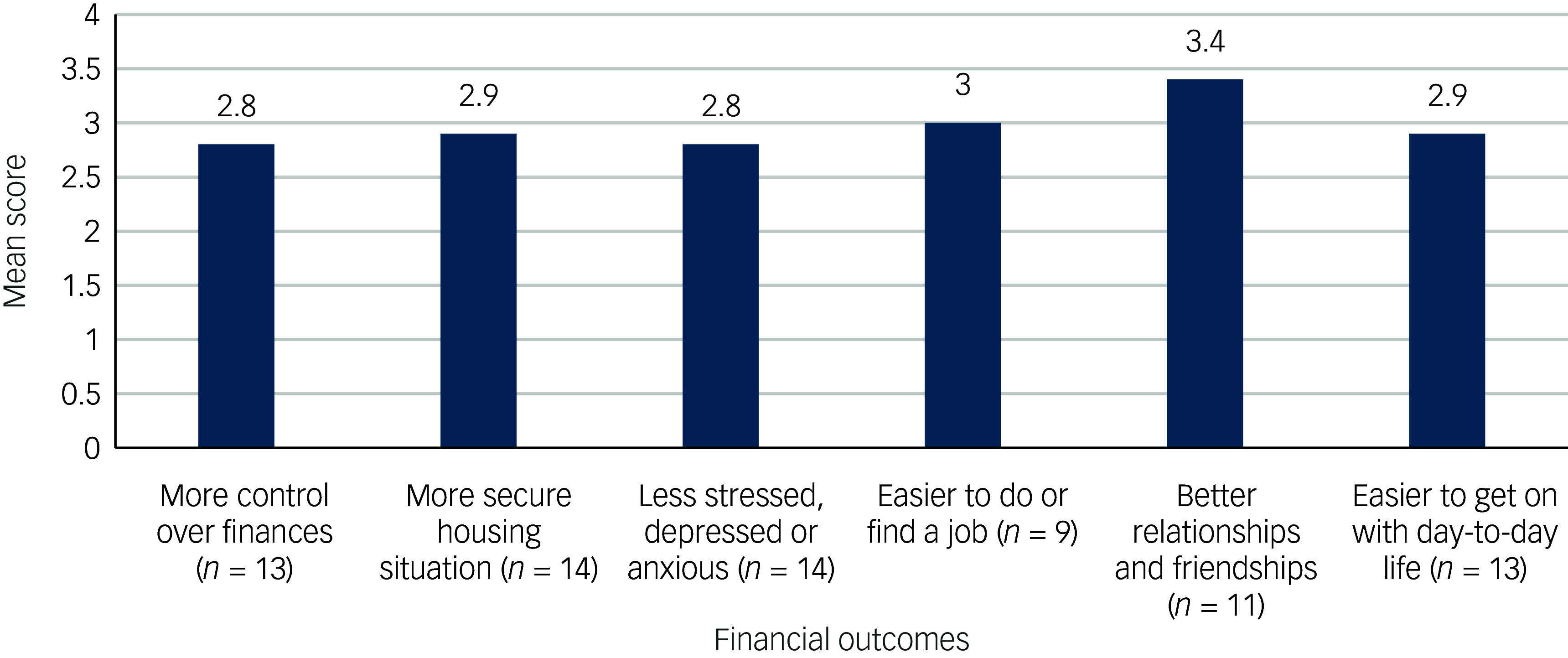
Mean scores for each item on the financial outcomes measure.

Finance scores (*n* = 14): The mean scores on each financial question were similar, on average between 2.8 and 3.4, with patients stating they had had modest improvements in areas of their lives relating to their financial situations at the end of psychological therapy. Not all the outcomes were relevant to all of these participants (see Fig. 1 for details).

#### Qualitative analysis

**Fig. 2:**
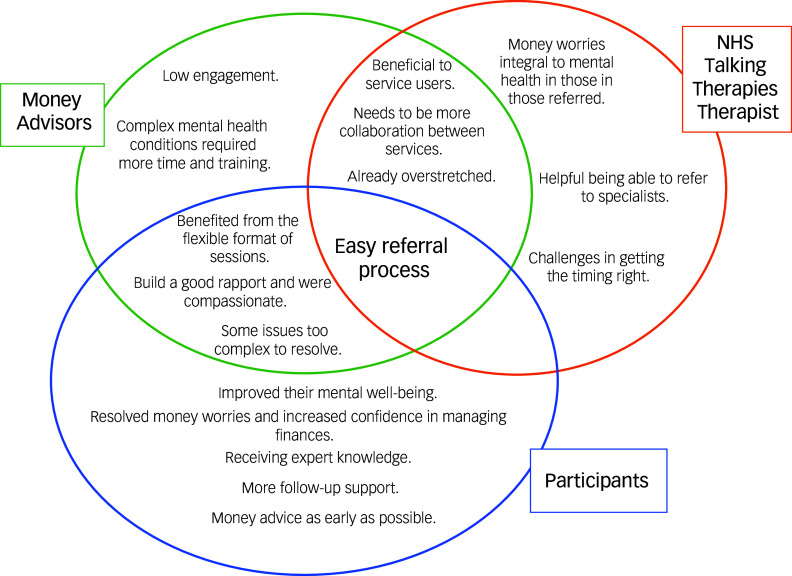
Coding overlap. NHS, National Health Service.

**Acceptability and feasibility:** Participants spoke about the acceptability and feasibility of the combined intervention, such as the processes involved in the referral and uptake of the money advice alongside psychological therapy (see Fig. 2 for the coding for each group of participants and Table 3 in the supplementary material for example quotes). Illustrative quotes are provided in Table 2.

**Table 2:** Example quotes from interviews with patients, therapists and money advisors

Theme	Subtheme	Patients	Therapists	Money advisors
Acceptability and feasibility	Money advice as early as possible	‘It should have been introduced earlier, I feel like it kind of prolonged some of the struggles and difficulties that I was having in therapy … I wasn’t getting the help in that department until the very end so if it was like introduced earlier it would be helpful.’		
	Overwhelming	‘I think irrespective of whether it was at the beginning or now I just think that when given information it can be like really overwhelming because a lot of content kind of like on board.’		
	Referral process was easy	‘I think the referral I found it was quick … it’s [a quicker] response than if you self-referral.’	‘I thought it was really straightforward. I had to think I had to follow up with them a couple of times to make sure they were sending the consent form, so that took a bit of time but on my end it was really straightforward.’	
	Money worries integral to mental health		‘I suppose all the clients that I have referred actually did quite well in therapy and kind of their social and financial circumstances were kind of quite instrumental to their worries.’	
	Challenges in getting the timing right		‘I think this is the problem. I think on a couple of occasions [I] will have asked him about the referral and whether he’d heard from them and whether things had started, and I think the response I got was, yes, I’ve heard from them and we’re trying to get started. [But] I don’t they really got started by the time we finished.’	
	Needs to be more collaboration between the services		‘It would have been helpful maybe to get an update, you know, has the person been accepted and what interventions might be offered.’	‘All we got was the referral sheet and the information on there was vague for the most point.’
	Already too overstretched		‘I think part if it is on the therapist’s side … there’s often, especially in an assessment, already so many things you kind of ask about, so it’s easy to kind of slip.’	‘I am the only one, so yeah, resources were stretched thinly. And yeah, it would have been great to have somebody alongside me doing this because, with the greatest will in the world, I’m one person, and I’m only here twice a week as I say, and I’ve got multiple responsibilities. So it’s, it’s tricky.’
	Complex mental health conditions required more time and training			‘When there was a client who was very difficult I would have liked to have a heads up … because I’m not a therapist, and I think in that specific example that client kind of felt what I was an extension of that service [Talking Therapies].’
	Low engagement			‘I think she said she had something else going on at the same time. Some other support, and so she felt like it was too much.’
Treatment benefits	Improved their mental well-being	‘I think it helps with like just routine and structure, so like having a phone call like weekly that you know you’re getting, I think that’s also kind of helped with I don’t know low mood and stuff.’	‘We had a treatment review after our support had ended and he mention that was a really big factor (money advice) in how he was feeling and why he was feeling better.’	
	Resolved money worries and increased their confidence in managing finances	‘getting the right help in terms of all these letters [coming] in and all that and having somebody to help me is something that, well, relieved me a lot. And having some solutions to certain things, yeah.’		
	Receiving expert knowledge	‘She was very, like well, [knowledgeable] in her field. And I had a lot of questions which she answered quite clearly.’		
	Benefited from the flexible format	‘Personally for me it was good that it was over the telephone … my schedule is a bit up in the air, so sometimes trying to go face to face with something is not always like positive if you see what I mean, because it’s just about trying to find that time and obviously if it’s over the telephone it’s a half an hour call, it’s super easy for me.’		‘So when she first came she said it was good for her to do this because she didn’t get out as much as she’d like. And so, you know that was positive.’
	Build a good rapport and were compassionate	‘He was quite nice, very friendly, very kind … he’s put me at ease.’		‘It’s been good for him to have that contact and be heard. And over the course of doing the PIP application like there being an understanding of what his experiences are, I feel like that was quite useful for him.’
	More follow-up support	‘I don’t know maybe there was kind of a limit to the consultation that [I] was supposed to have because … we don’t have more than three consultations … I would have appreciated that he actually bring the options and discuss the options with me because it’s more or less like he just gave me the options and go off and then say like you know if you need any more help you [can] come back to me.’		
	Some issues were too complex to resolve	‘I feel like the advice I was given to me was focused on debt, which is a large part of the money issues but it’s just a part and that’s where like um income that wasn’t covered at all, I wasn’t given like advice and tips on um how I can improve my income or like my income streams.’		‘we are limited to a certain extent [in] the advice that we can give and support so, some cases when it’s a little bit above what we can do, we would advise them to contact a solicitor if they can afford, for example, in this case, if we’re discussing about a repossession of a property solicitor has … but again, that’s subject to money, because they’re not free, so sometimes they might just find advice somewhere else, or they decide to take different sort of action and then maybe engage but it depends from case to case.’
	Helpful being able to refer to specialists		‘We can put plans into place and more of a specific sort of mental health capacity rather than focusing entirely on financial difficulties, so I found it really useful.’	

PIP, Personal Independence Payment.

Patients reported that they wanted the money advice as early as possible, with many saying it came too late and would have been more helpful if introduced earlier. However, they also reported that getting money advice was somewhat overwhelming, which led to some missing sessions or putting off important actions. They also said that the referral process was easy and that the response to it was quick, suggesting that referral was acceptable.

Therapists felt money worries were integral to the mental health of those referred, indicating that the service was suitable for those most in need and provided important additional support. The referral process was easy, but there were challenges in getting the timing right as the combined intervention meant the two services ideally run in parallel, but often therapists were not aware of financial difficulties until later in treatment or the client did not immediately consent and follow up the referral. More collaboration between the services was requested, such as being updated on their client’s money advice process, suggesting challenges when the services were independent. However, therapists also reported that they were already too overstretched and did not check in with the money advisors or delve into financial difficulties unless they came up within therapy.

Money advisors agreed that the referral process was easy and that more collaboration between the services was required but, like the psychological therapists, that they also were already too overstretched. In addition, they commented that patients presenting with complex mental health conditions required more time and training. They often referred to these patients as being ‘difficult’ and being unsure about their diagnoses or how best to approach them. They reported that clients had low engagement, which they thought was caused by these individuals struggling to communicate with them and not turning up to appointments.

#### Treatment benefits

Therapists spoke about how helpful it was to refer patients to, and for patients to receive, money advice alongside psychological therapy, as well as the ways in which it was helpful (see Fig. 2 and Table 2).

Patients reported that receiving the combined intervention improved their mental well-being, for example, it gave them a sense of relief and provided them with a structure that helped battle their low mood. The money advice helped them resolve money worries and increase their confidence in managing finances. For them, receiving expert knowledge from the money advisors provided more targeted support. They found aspects of the money advice particularly beneficial, such as the flexible format that was personalised to their needs and was convenient. They also found it important that money advisors could build a good rapport and were compassionate to their situations. Some felt the money advice was too short, and they would have appreciated more follow-up support after the initial consultation and advice was given as some issues were likely to take a longer time to resolve. Some also felt their difficulties lay outside the remit of the money advisors and were too complex to resolve or advice would focus on debt but no other contributing factors, such as generating income or legal issues.

Therapists agreed that the money advice was beneficial to clients as it improved their mental well-being and clients told them that it had helped. They also found it helpful being able to refer to specialists, as money problems were often part of clients’ presentations but could not be resolved within the therapy and were outside the therapist’s remit.

Money advisors agreed that the money advice was beneficial and that the clients benefited from the flexible format. For example, money advisors saw advantages in having both face-to-face and virtual/telephone appointments, and that they could build a good rapport and were compassionate towards the clients while giving advice. However, they also reported that some issues were too complex to resolve or lay outside their remit.

## Discussion

### Combined intervention success

The study was carried out in areas of high deprivation and attracted a diverse group of participants, with almost half identifying as being from an minoritised background. Previous research has found that this group was at an increased risk, with individuals from ethnic minority backgrounds being 2.5 times more likely to be in poverty than those from White backgrounds,^10^ being disproportionally affected by the current cost-of-living crisis^27^ and less likely to access financial advice.^28^ The intervention attracted more women (68.6%), which is representative of the clients in Talking Therapies services,^26^ but may also reflect previous findings of women being at a higher risk of financial difficulties (e.g. Tims and Caddick^9^).

Our findings demonstrate that 91% of those referred were able to complete at least one session with a money advisor, indicating an acceptable referral process, which was also demonstrated in all our qualitative interviews. This also shows a high level of motivation to engage with the service. This is much higher than adherence rates to Talking Therapies services by itself, where in 2020–2021 69.9% of those referred to Talking Therapies attended their first session.^16^ In addition, a systematic review of adherence measures in over 76 intervention randomised control trials demonstrated an average adherence rate of 80.8%.^29^ This study also faced an additional barrier to attendance as it was completed over the COVID-19 period when patient contact in general was a challenge.

Our findings showed promising benefits. A third moved to recovery on symptoms of anxiety, 40% on symptoms of depression and a third to recovery on both measures. This is greater than the previously estimated recovery rates for individuals with depression and money worries (22%).^14^ Only 20% of our patients moved to recovery on levels of functioning, although there are no benchmarks for this figure using the WSAS-5. Half our participants demonstrated an improvement on both the GAD-7 questionnaire and PHQ-9 despite the seriousness of their mental health problems showing a clear need for high-intensity services. Our participants might also have different therapeutic goals than symptom ‘recovery’, such as being more confident in their finances, and this might be an outcome driven by only a small change in anxiety or depression and vice versa. In future, these combined interventions might adopt different measures, such as the Goal Attainment Scale,^30^ which considers the most immediate and valued personal goals as set by patients themselves. Financial outcomes measures showed only modest levels of confidence, but even these levels are an improvement, as everyone in the sample had complained about having money worries. Participants also had difficulties that lay outside the money advisor’s remit, indicating the complex needs that would benefit from a multi-agency approach.

We had hoped for a joint service,^11^ but the two services operated under different guidelines and procedures and were separate geographically. For example, advisors from Citizens Advice must be regulated by the Financial Conduct Authority. We tried to facilitate collaboration by gaining the patient’s consent for the two agencies to discuss their case; however, this did not happen. A shared notes system is not possible within the current procedures. However, a future combined intervention service should try to collocate services, as the Money and Mental Health Policy Institute (MMHPI) is urging NHS England to do.^31^ Another issue we identified was the timing of the intervention, with patients wanting to receive money advice as early as possible, but therapists found that identifying this need early enough for a timely referral was a challenge. The MMHPI has also recognised this issue, suggesting that NHS Talking Therapies should include a screening question at assessment to identify patients who would benefit. Lastly, the current study was unable to ascertain the level and types of financial difficulties experienced, as referrals to the money advice intervention were based on participants’ subjective need for help with money worries that were affecting their mental health. To investigate whether a combined intervention is effective, it will be important for future studies to conduct randomised control trials that also examine whether the type and level of money worries affects the outcomes of the intervention.

### A future service

Both agencies that create the combined service must have enough resources allocated to fulfil all key points in the combined intervention. This would also ensure early identification of money worries, a smooth referral process, training for money advisors to work with complex mental health needs, timely provision of the money advice service and more contact between the two agencies.

Whilst it is beyond the scope of the current paper, our findings did suggest that the complexity of the cases may have affected the benefits of the psychological treatment and financial advice given. NHS Talking Therapies currently uses a stepped care approach, and we only looked at patients who had reached the highest level of stepped care. However, there have been recent calls to integrate fragmented healthcare systems by screening patients more comprehensively and stratifying them to treatments that are best suited for their problems.^32,33^ A future service may benefit from using a more stratified approach with new referrals who have financial difficulties being referred to a joint money advice and psychological treatment to assess whether this combined approach is also helpful for a less symptomatic group.

In conclusion, our findings indicate the importance and acceptability of a combined money advice and psychological therapy service, which is accessible to those most in need (e.g. those at high risk of financial problems and those whose money worries have affected their mental health). By assessing intervention feasibility, we have identified strategies that will allow the two services to integrate and encourage multi-agency collaboration: (a) a policy for NHS Talking Therapies services to include screening questions on money worries as well as collect additional outcome measures and (b) securing adequate resources to fund money advisors’ time and training. Our initial assessment suggested that the combined intervention could be effective; a future randomised study could compare outcomes of those receiving the combined intervention with those receiving only psychological therapy perhaps with signposting to money advice. This trial would be feasible given our diverse recruitment and high take-up of the referral system, ease of data collection and signs of benefit. This trial would show the extent of the benefit to recovery. However, given the known effects on money worries on recovery outcomes, it may be more ethical to implement this combined service to counter the deprivation effects^1^ perhaps as part of a stepped wedge trial. Further partnership working between the two services would also increase the combined intervention effectiveness.

## Supporting information

Belcher et al. supplementary material Belcher et al. supplementary material

## Data Availability

The data that support the findings of this study are available on request from the corresponding author, H.L.B. The data are not publicly available as they may contain information that could compromise the privacy of research participants.

## Supplementary material

The supplementary material is available online at https://doi.org/10.1192/bjo.2025.37
