# Supplementary material for: Feasibility and potential effects of a combined money advice and psychological therapy intervention within National Health Service Talking Therapies services
Source: BJPsych Open. 2025 Jun 11;11(4):e120. doi: 10.1192/bjo.2025.37 (PMC12188236; doi:10.1192/bjo.2025.37)
Supplement: Belcher et al. supplementary material [file S2056472425000377sup001.docx]

**Supplementary Information**

**1. Topic Guide – Service Users**

**Background information:**

- Are you still receiving support from the money advisor?
  - If no, did you complete your planned sessions with the money advisor? If not, how come?
  - How many money advice sessions did you received in total?
- Did you complete your planned therapy sessions with your IAPT therapist? If not, how come?
- How many IAPT therapy sessions did you receive in total?

**In depth questions:**

1. How did you find receiving this money advice as part of your IAPT treatment?

- Prompts: Was it helpful, were you able to engage in both, was the support useful, what effect did it have on your mental health and therapy, what effect did it have on your money worries

1. How did you find the referral process to the money advisor?

- Prompts: Was it straightforward, were you given enough information, when in your treatment were you referred, was this introduced at the right time and in the right way

1. How did you find the way that the money advice was delivered?

- Prompts: Did you receive the support in a format you wanted, was it easy to understand, were the frequency and length of sessions adequate, were you able to follow the advice given, was it accessible to your needs, did it integrate with your therapy

1. Did you encounter any problems with taking part in both money advice and IAPT therapy?

- Prompts: Timing, format/delivery, therapy specific issues, money advice specific issues, unresolved money worries

**Topic Guide – Staff**

**Background information:**

- How many clients did you see that were receiving both IAPT therapy and money advice?
- How many sessions did you deliver to these clients?
- Did they complete their planned number of sessions?
- If not, why not?

**In depth questions:**

1. How did you find providing your patient(s) with support on this combined intervention?

- Prompts: engagement, benefits to clients, effectiveness, effect on mental health, effect on money worries

1. How did you find the process of referring patient(s)?

- Prompts: was it straightforward, how much time did it take, how easy was it introducing the subject of money worries, was the referral comprehensive enough, was more information needed, how long did it take to schedule a first meeting after referral was made

1. How acceptable did you find the way this combined intervention was delivered?

- Prompts: did you think the format was appropriate, was there enough time to resolve clients problems, how much time was needed, were the length of sessions adequate, were clients able to follow the advice given, did it integrate well with the other service

1. How did you find working collaboratively with the other service?

- Prompts: how regularly did you meet/speak, were you able to discuss cases when needed, was sharing information straightforward, did clients find this helpful, did you receive adequate training

1. Did you encounter any problems with the combined intervention?

- Prompts: Timing, format, therapy specific issues, money advice specific issues, unresolved money worries, time burden, resources, integration of services

**Consort Diagram**

Assessed for eligibility = 73

Enrolment

Routine NHS Talking Therapies data = 29

- Excluded from some analysis = 2 (1 did not complete baseline measure of WSAS-7, 1 fell below cut off criteria on GAD-7)

End of treatment study follow-up = 14

Analysis

Lost to routine NHS Talking Therapies questionnaire follow-up = 1 (did not complete any measures)

Lost to end of treatment study follow-up = 18 (did not attend end of treatment interview)

Follow-up

Excluded = 3

- Did not attend intervention

Received intervention = 32

Allocated

Excluded = 38

- Declined to participate/did not provide written consent - 38

**Table 3. Individual service-user pre- and post- GAD-7 and PHQ-9 scores, significant improvements and reliable recovery**

| Participant | Pre-GAD | Post-GAD | GAD recovery | Pre-PHQ | Post-PHQ | PHQ recovery | Overall recovery | Pre-WSAS | Post-WSAS | WSAS Recovery |
| --- | --- | --- | --- | --- | --- | --- | --- | --- | --- | --- |
| 1 | 13 | 3 | Yes | 20 | 8 | Yes | Yes | 23 | 16 | No |
| 2 | 21 | 19 | No | 26 | 25 | No | No | 32 | 30 | No |
| 3 | 15 | 1 | Yes | 18 | 2 | Yes | Yes | 21 | 6 | Yes |
| 4 | 17 | 15 | No | 10 | 15 | No | No | 2 | 13 | No |
| 5 | 19 | 7 | Yes | 20 | 10 | No | No | 29 | 3 | Yes |
| 6 | 7 | 2 | N/A* | 18 | 4 | Yes | Yes | 23 | 8 | Yes |
| 7 | 15 | 17 | No | 20 | 17 | No | No | 21 | 25 | No |
| 8 | 10 | 14 | No | 15 | 19 | No | No | 18 | 21 | No |
| 9 | 18 | 12 | No | 18 | 9 | Yes | No | 26 | 19 | No |
| 10 | 15 | 21 | No | 21 | 27 | No | No | 28 | 31 | No |
| 11 | 14 | 8 | No | 11 | 4 | Yes | No | 19 | 23 | No |
| 12 | 16 | 21 | No | 20 | 27 | No | No | 24 | 35 | No |
| 13 | 12 | 13 | No | 20 | 21 | No | No | 35 | 31 | No |
| 14 | 15 | 2 | Yes | 18 | 3 | Yes | Yes | 23 | 17 | No |
| 15 | 10 | 15 | No | 19 | 20 | No | No | 33 | 15 | No |
| 16 | 12 | 16 | No | 24 | 24 | No | No | 35 | 32 | No |
| 17 | 12 | 13 | No | 10 | 11 | No | No | 26 | 30 | No |
| 18 | 15 | 2 | Yes | 14 | 1 | Yes | Yes | N/A | 2 | N/A |
| 19 | 10 | 10 | No | 14 | 13 | No | No | 24 | 25 | No |
| 20 | 13 | 14 | No | 19 | 14 | No | No | 36 | 23 | No |
| 21 | 18 | 12 | No | 19 | 17 | No | No | 20 | 24 | No |
| 22 | 20 | 13 | No | 23 | 12 | No | No | 27 | 24 | No |
| 23 | 19 | 13 | No | 23 | 15 | No | No | 9 | 33 | No |
| 24 | 12 | 3 | Yes | 17 | 4 | Yes | Yes | 11 | 10 | No |
| 25 | 16 | 4 | Yes | 21 | 5 | Yes | Yes | 11 | 21 | No |
| 26 | 21 | 20 | No | 24 | 19 | No | No | 26 | 28 | No |
| 27 | 17 | 3 | Yes | 26 | 9 | Yes | Yes | 29 | 10 | No |
| 28 | 9 | 1 | Yes | 13 | 0 | Yes | Yes | 20 | 1 | Yes |
| 29 | 15 | 7 | Yes | 15 | 9 | Yes | Yes | 24 | 14 | No |
| 30 | 13 | 10 | No | 15 | 12 | No | No | 20 | 12 | No |
| 31 | 17 | 19 | No | 12 | 16 | No | No | 19 | 19 | No |
| 32 | N/A | N/A | N/A | N/A | N/A | N/A | N/A | N/A | N/A | N/A |

* Indicates individuals who did not reach symptoms threshold at pre-assessment
